# Plasma cardiac troponin T complements neurofilament light chain by reflecting disease phase and phenotypic variation in amyotrophic lateral sclerosis

**DOI:** 10.1007/s00415-026-14002-w

**Published:** 2026-07-27

**Authors:** Julia Sellin, Linn Öijerstedt, Janina von der Gablentz, Sanharib Chamoun, Ulf Kläppe, Rayomand Press, Kristin Samuelsson, Julian Grosskreutz, Caroline Ingre

**Affiliations:** 1 Precision Neurology of Neuromuscular and Motor Neuron Diseases, University of Lübeck, Lübeck, Germany; 2 Department of Clinical Neuroscience, Karolinska Institute, K8 Neuro Ingre, 171 77 Stockholm, Sweden; 3 Department of Neurology, Karolinska University Hospital, Stockholm, Sweden; 4 Cluster for Precision Medicine in Inflammation, Universities of Lübeck, Lübeck, Germany

**Keywords:** Amyotrophic lateral sclerosis, Troponin-T, Neurofilament, Disease aggressiveness, Progression, D50-Model

## Abstract

**Background:**

Neurofilament light chain (NfL) is an established marker of neuronal injury and disease aggressiveness in amyotrophic lateral sclerosis (ALS). In contrast, the clinical and biological significance of cardiac troponin T (cTnT) in ALS is not fully understood. We aimed to evaluate the relationship between plasma cTnT and disease aggressiveness, progression stage, and clinical phenotype in comparison with NfL.

**Methods:**

Plasma cTnT and cerebrospinal fluid (CSF) NfL were analysed at diagnosis in a population-based cohort of 526 patients with ALS. Disease aggressiveness was modelled using the D50 framework, which quantifies the time taken to lose 50% of functional capacity (ALSFRS-R) and normalises individual disease trajectories. Biomarker associations with disease aggressiveness, phase, and clinical variables were assessed through group comparisons, logistic and linear regression, and receiver operating characteristic analyses.

**Results:**

Neurofilament light chain in CSF was strongly associated with disease aggressiveness, with higher levels in patients with more aggressive disease. In contrast, plasma hs-cTnT did not correlate with D50. Across disease phases, CSF NfL remained relatively stable, whereas plasma hs-cTnT increased with advancing rD50, suggesting accumulation with disease progression. Plasma hs-cTnT levels were higher in patients with spinal compared to bulbar onset. Combined biomarker models improved sensitivity and negative predictive value for identifying less aggressive disease.

**Conclusion:**

Plasma hs-cTnT and CSF NfL capture distinct but complementary dimensions of ALS pathology. While NfL reflects disease aggressiveness, hs-cTnT aligns with disease phase and clinical phenotype, supporting its use as a complementary biomarker for ALS characterisation and monitoring.

**Supplementary Information:**

The online version contains supplementary material available at 10.1007/s00415-026-14002-w.

## Introduction

Amyotrophic lateral sclerosis (ALS) is a progressive neurodegenerative disorder with considerable clinical and biological heterogeneity. Over the past decade, neurofilament light chain (NfL) measured in cerebrospinal fluid (CSF) has emerged as a leading biomarker in ALS. Numerous studies have demonstrated that elevated CSF NfL levels are associated with faster functional decline and shorter survival in ALS [1, 2]. However, NfL primarily reflects central neurodegeneration and provides limited information about processes occurring downstream in muscle tissue or the neuromuscular junction [3, 4]. This leaves an unmet need for biomarkers that complement NfL by capturing other facets of ALS biology. One promising candidate is cardiac troponin T (cTnT), a biomarker traditionally used to detect myocardial injury but increasingly recognized for its potential relevance in neuromuscular disorders [5, 6]. We previously demonstrated that plasma cTnT is elevated in patients with ALS compared to healthy controls [3, 7]. In addition, recent work from our group shows that high plasma cTnT is associated with muscle denervation, further positioning it as a marker of peripheral muscle involvement in ALS [8, 9]. Despite these emerging observations, the clinical significance of cTnT in ALS remains unclear. In particular, little is known how plasma cTnT relates to disease aggressiveness or clinical phenotype, or how it compares to CSF NfL as marker of disease severity.

The D50 model of disease progression was developed to address the non-linearity of ALSFRS-R progression rate and its inherent low signal to noise ratio. The model uses iterative fitting of all available ALSFRS-R scores for every individual to empirically determine time constant and turning point (D50) of a sigmoidal curve from total health to functional loss [10, 11]. Heterogeneous progression patterns become comparable by capturing both global disease aggressiveness (D50) and relative disease phase (rD50), representing the proportion of individual disease covered. The D50 model has validated a range of different biomarker signals including their capability to predict functional loss rather than survival[12–16].

The aim of this study was to evaluate the value of plasma cTnT in relation to disease aggressiveness and clinical phenotype, particularly in comparison to CSF NfL. We hypothesised that troponin, as a suggested marker of muscle damage, contributes additional clinical insight beyond what is captured by neurofilament light chain alone.

## Material and methods

### Cohort and data collection

A total of 526 consecutive patients diagnosed with ALS were recruited at the Neurology Clinic, Karolinska University Hospital, Stockholm, Sweden, between November 2014 and November 2024. All patients fulfilled the Gold Coast diagnostic criteria for ALS. During this period, each patient underwent clinical evaluation and provided blood and/or CSF samples as part of the diagnostic work-up. Collected clinical data included sex, site of symptom onset, and presence of a known genetic cause of ALS. Functional impairment was assessed using the revised ALS Functional Rating Scale (ALSFRS-R). The ALSFRS-R derived progression rate (DPR) was also utilised for the purpose of comparison, by subtracting ALSFRS-R score obtained at the time of sampling from 48 (maximum score), divided by the time in months from onset to this ALSFRS-R assessment, with subgroups classified as follows: fast (DPR ≥ 1.5), medium (0.5 ≤ DPR < 1.5) and slow (DPR ≤ 0.5) (Meyer et al., 2025).

All participants provided informed consent prior to inclusion in the study. Ethical approval was obtained from the Swedish Ethics Review Authority (diary number 2017-1895-31-1), and all procedures were conducted in accordance with the Declaration of Helsinki.

### Sample collection and protein analysis

Venous blood and/or CSF samples were collected at the time of diagnosis during the recruitment period (November 2014–November 2024). Plasma concentrations of high-sensitive cardiac troponin T (hs-cTnT) and NfL in serum and CSF were analysed at the Karolinska University Laboratory, a fully accredited clinical laboratory. Plasma hs-cTnT was measured using the Elecsys assay (Roche Diagnostics, Rotkreuz, Switzerland). NfL was quantified using a sandwich enzyme-linked immunosorbent assay (ELISA) from UmanDiagnostics (Umeå, Sweden; catalog number 10–7001).

Within individuals, biomarker data were paired only for analyses directly comparing CSF NfL and plasma hs-cTnT. Each CSF NfL measurement was matched to the temporally closest hs-cTnT value within the same individual. NfL and hs-cTnT sampled within 6 months (*N* = 302) were considered temporally matched, whereas pairs sampled more than 6 months apart (*N* = 224) were retained in the analysis to maximize sample size. The time difference (in days) between paired measurements was recorded. Analyses evaluating each biomarker separately used all available measurements irrespective of pairing.

### D50 disease progression model

**Fig. 1:**
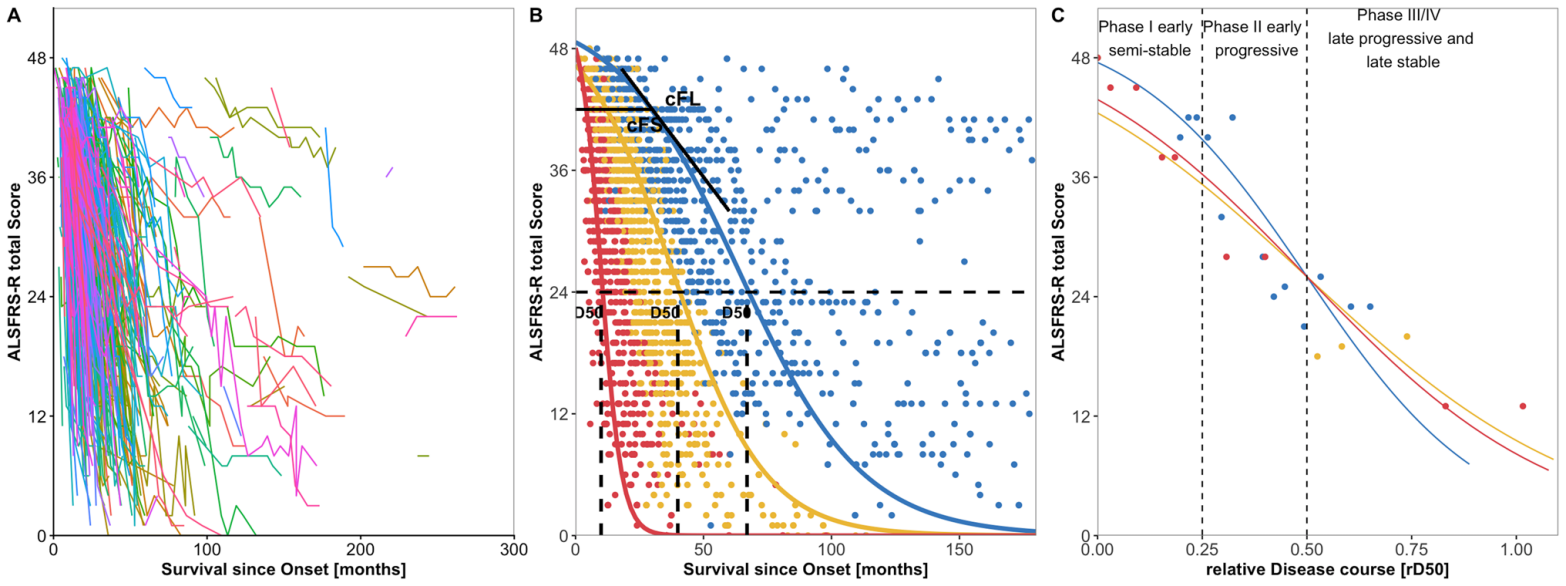
D50-modeling of disease progression (*n* = 526). **A** ALSFRS-R total scores over time of survival from onset in months (*n* = 526) with all individual scores per patient. **B** ALSFRS-R total scores over the time of survival from symptom onset in months with indicated D50-values (timepoint of 50% loss of motor function). **C** Normalizing real time months to the individual D50 values translates disease trajectory rD50, showing that disease aggressiveness subgroups go through the same form of functional decline during their individual disease course, and calculated for each ALSFRS-R sampling date with one representative patient per disease aggressiveness subgroup. Red = high (D50 ≤ 20 months), yellow = intermediate (20 < D50 < 40 months), blue = low aggressive disease (D50 ≥ 40 months)

The D50 model of ALS progression provides a quantitative measure of disease aggressiveness that is distinct from disease accumulation to interpret biomarker signals [10, 12, 17]). The model calculates an individual sigmoidal fit from functional health to loss of motor function, based on all available ALSFRS-R scores longitudinal collected on a regular basis for each individual patient (Fig. 1A). The sigmoidal fit is described with two parameters: (1) D50 as time in months since symptom onset to timepoint of 50% motor function loss, and (2) dx as the time constant of functional decline, i.e. steepness of the curve (Fig. 1B). D50 and dx demonstrate a strong linear correlation shown in several different cohorts ([11, 12, 17]) which was confirmed for this cohort (Supplementary Fig. 2; *R*^2^ = 0.94, *p* < 0.001). Thus, only D50 is utilised to describe the disease aggressiveness exhibited by an individual patient. The participants in this study were divided into three subgroups based on their D50-derived disease aggressiveness: high (D50 < 20 months), intermediate (20 ≤ D50 < 40 months), and low (D50 ≥ 40 months). A second step normalises real-time observations since symptom onset in months to the D50 value, which results in the calculation of the relative D50 (rD50) parameter. The rD50 parameter generates an open-ended scale, with 0.5 representing the D50 value and 0 representing the disease onset (Fig. 1C). This allows the calculation of rD50 for any given observaton, and it is independent of the overall aggressiveness of the disease. Furthermore, it provides an individualised quantification of disease accumulation. The rD50 parameter is employed to categorise patients according to the progression patterns exhibited during the course of their disease. The categorisation system comprises three distinct phases (Fig. 1C): Phase I, designated as the early semi-stable phase, is characterised by rD50 values ranging from 0 to 0.25. Phase II, designated as the early progressive stable phase, is characterised by rD50 values ranging from 0.25 to 0.5. Phase III/IV, designated as the late progressive and stable phase, is characterised by rD50 values beyond 0.5. The D50 model in addition provides descriptors of local disease activity at any given time point with a precision at least 5 times higher than the raw ALSFRS-R values at or near this time point and its derivative DPR. These include the calculated functional loss rate (cFL) and the calculated functional state (cFS) [12].

### Statistical analysis

The statistical analysis and graphical visualisation of the data were conducted using RStudio open-source software (Version 2023.06.0, Posit Software, PBC, Boston, MA, United States) and the R programming language on a macOS Sequoia 15.5. All visualizations were performed with the additional packages *ggplot2, ggpubr, ggbeeswarm* and *ggthemes*. Normality was assessed through the Shapiro–Wilk test. Statistical analyses were conducted using the Kruskal–Wallis test for continuous, non-normally distributed variables (post hoc test with Wilcoxon rank sum test), and Pearson’s chi-square test for categorical data (*gtsummary*). The correlation between CSF NfL and plasma hc-cTnT was examined using Spearman’s rank correlation (*stats*)*.* In a subset of 109 patients with paired CSF and serum samples collected on the same day, the association between CSF and serum NfL concentrations was assessed using Pearson’s correlation and linear regression.

Linear regression models were applied to assess the relationship between biomarkers and disease aggressiveness and disease phase (*stats*)*.* The models included D50 or rD50 as the outcome variable, CSF NfL or plasma hs-cTnT as the predictor, and separated by disease aggressiveness. All variables were log_n_ transformed prior to modelling to improve model fit and meet distributional assumptions.

To evaluate the discriminatory power of CSF NfL, plasma hs-cTnT, and their combined ability, logistic regression models were constructed using disease aggressiveness (high vs low D50, *N* = 177) as outcome (*stats*)*.* Three models were fitted: one including CSF NfL alone, one including plasma hs-cTnT alone, and one including an interaction term between CSF NfL and plasma hs-cTnT. Models were adjusted for age at symptom onset, sex, site of symptom onset, and rD50 phase. The discriminatory performance of each model was evaluated using receiver operating characteristic (ROC) curves, the area under the curve (AUC), sensitivity, specificity, positive predictive value (PPV), and negative predictive value (NPV) (*pROC*)*.* Similarly, logistic regression models were applied to predict site of symptom onset (bulbar vs spinal). These models were adjusted for D50 (high, intermediate, low), age at symptom onset, sex, and rD50 phase.

To evaluate the prediction of D50 from NfL, hs-cTnT and their combination (NfL + hs-cTnT), two different modelling methods were used, linear regression model and non-linear regression model (*gslnls)*. Model accuracy was assessed with the function *goodness_of_fit* from the *chemdeg* package. Ultimately, we obtain disparate “goodness of fit” values for the non-linear regression models, with the Akaike information criterion (AIC) and Root Mean Square Error (RMSE), which quantify the relative quality of statistical models for a given set of data. For the linear model, the R^2^ and adjusted R^2^ metrics were also used to evaluate model fit.

A *p*-value of < 0.05 was considered statistically significant. Post-hoc comparisons were adjusted for multiple testing using the Bonferroni correction.

## Results

### Cohort

**Fig. 2:**
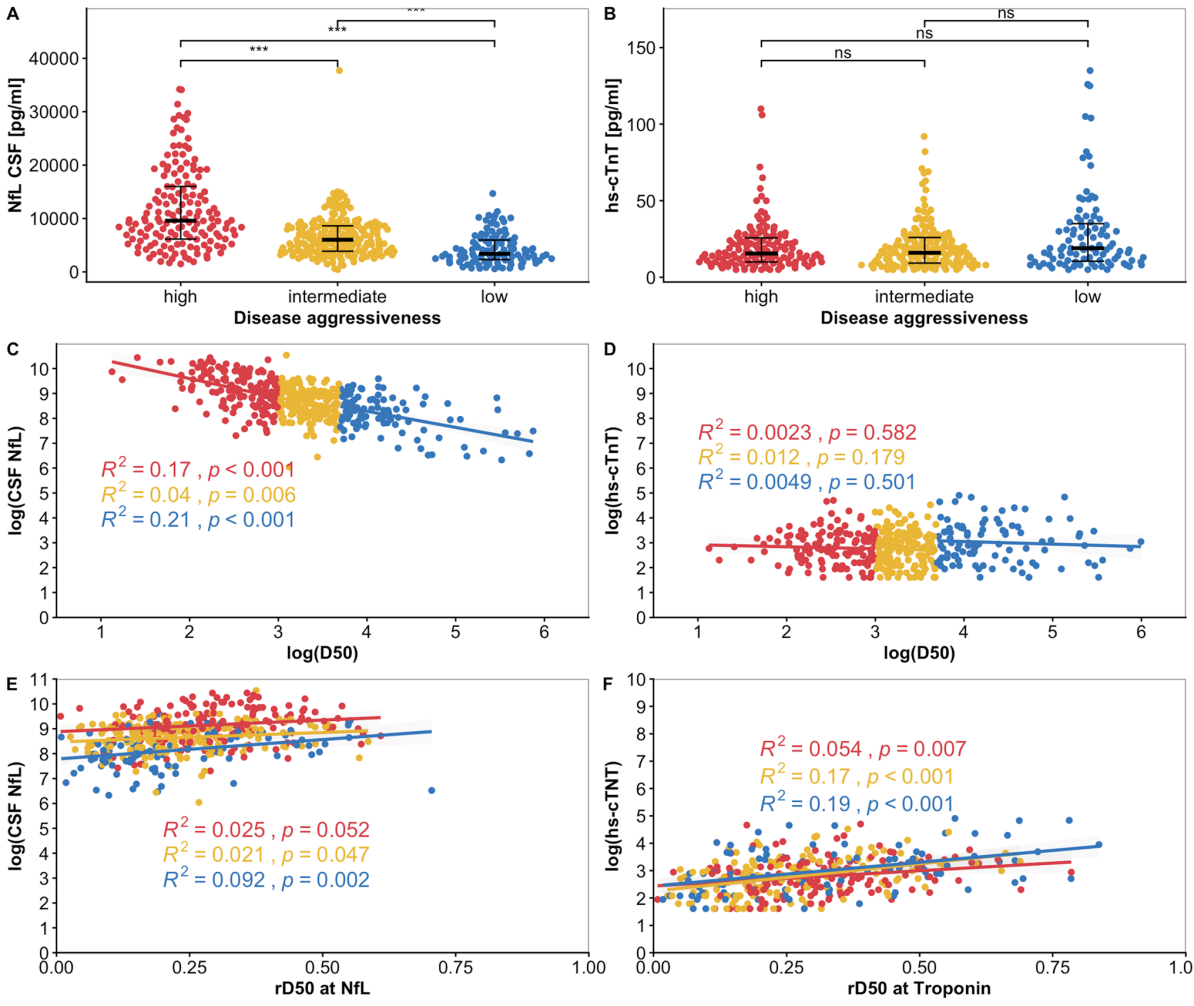
Relationship between biomarkers at the time of ALS diagnosis and disease aggressiveness (*n* = 526). **A** NfL in CSF by disease aggressiveness **B** hs-cTnT in plasma by disease aggressiveness, **C** Linear regression with CSF NfL levels by D50, **D** Linear regression with plasma hs-cTnT levels by D50, **E** Linear regression with CSF NfL by rD50 at the time of sampling, **F** Linear regression with plasma hs-cTnT by rD50 at the time of sampling. In A and B, median values are indicated as horizontal lines and IQR as error bars, Wilcoxon-Rank-Sum-Test: *** = *p* < 0.001, * = *p* < 0.05, ns = non-significant; colors: D50-derived disease aggressiveness, red = high (D50 ≤ 20 months), yellow = intermediate (20 < D50 < 40 months), blue = low aggressive disease (D50 ≥ 40 months)

The clinical and demographic data of the 526 patients is presented in Table 1, with the participants divided into subgroups based on disease aggressiveness as determined by D50. Patients with high disease aggressiveness were older at symptom onset (median age 69 vs 62 years in the high and low group respectively, *p* < 0.001) and had a shorter time from symptom onset to the first ALSFRS-R assessment (*p* < 0.001). Bulbar onset was more common in the high aggressiveness group (35%) compared to the low group (23%), although the difference in onset site distribution did not reach statistical significance (*p* = 0.087). It is important to note that the disease accumulation on the day of the first ALSFRS-R score, measured via relative D50 (rD50), differed significantly between the three disease aggressiveness subgroups. This phenomenon represents sampling shift due to disease aggressiveness; it exists in all ALS cohorts but to our knowledge is quantified only in the D50 model. Consequently, the varying composition of strata in D50-derived disease phases substantiates the necessity to analyse disease accumulation and disease aggressiveness separately (Fig. 2 and suppl Fig. 4). The rate of disease progression, as measured by the ALSFRS-R score, also exhibits significant variability among subgroups of differing disease aggressiveness. When progression rate is calculated from the first available ALSFRS-R assessment, approximately 20–30% of patients are assigned to a different aggressiveness category than when using the D50 model (see also Meyer et al., 2025).

**Table 1:** Demographics and clinical data for patients with ALS, stratified into D50-derived disease aggressiveness groups (*n* = 526)

	Disease aggressiveness			*p* value
	High *N* = 175	Intermediate *N* = 215	Low *N* = 136	
Clinical characteristics				
No. of females	77 (44%)	105 (49%)	73 (54%)	0.2^a^
Age at symptom onset, median years (IQR)	69 (59, 74)	65 (56, 72)	62 (51, 70)	< 0.001^b*^
Time from symptom onset to first ALSFRS-R, median months (IQR)	8 (7, 11)	14 (11, 19)	38 (22, 75)	< 0.001^b*^
First ALSFRS-R score, median (IQR)	36 (30, 41)	40 (36, 43)	38 (29, 43)	< 0.001^b*^
Site of symptom onset, *N*				0.087^a^
Bulbar	62 (35%)	80 (37%)	31 (23%)	
Spinal	94 (54%)	115 (53%)	93 (68%)	
Other	11 (6%)	12 (6%)	8 (6%)	
Unknown	8 (5%)	8 (4%)	4 (3%)	
Genetics, *N*				
* C9orf72*	22 (13%)	18 (8%)	9 (7%)	
* SOD1*	1 (0.6%)	2 (1%)	11 (8%)	
Negative screening panel	90 (51%)	140 (65%)	87 (64%)	
Not screened	62 (35%)	55 (26%)	29 (21%)	
Disease progression rate^c^, median (IQR)	1.43 (0.98, 2.03)	0.60 (0.40, 0.89)	0.37 (0.18, 0.65)	< 0.001^b*^
Disease progression rate^c^ group, *N*				< 0.001^a*^
Fast	84 (48%)	9 (4.2%)	9 (6.6%)	
Medium	85 (49%)	146 (68%)	43 (32%)	
Slow	6 (3.4%)	60 (28%)	84 (62%)	
NfL, cTnT and D50 metrics				
D50 at baseline, median months (IQR)	14 (11, 18)	27 (23, 32)	63 (49, 98)	< 0.001^b*^
rD50 at baseline ALSFRS-R, median (IQR)	0.34 (0.26, 0.43)	0.27 (0.19, 0.36)	0.28 (0.17, 0.46)	< 0.001^b*^
Disease phase, *N*				< 0.001^a*^
I	40 (23%)	95 (44%)	62 (46%)	
II	110 (63%)	105 (49%)	47 (35%)	
III/IV	25 (14%)	15 (7%)	27 (20%)	
CSF NfL and cTnT sample status, *N*				
Matched NfL and cTnT	117 (67%)	141 (66%)	73 (54%)	
NfL only	37 (21%)	50 (23%)	27 (20%)	
cTnT only	21 (12%)	24 (11%)	36 (26%)	
CSF NfL, median pg/ml (IQR), *N* = 445	9 590 (6 140, 16 000)	6 020 (3 860, 8 630)	3 375 (2 265, 6 055)	< 0.001^b*^
Time from onset to NfL, median months (IQR)	7 (5, 11)	12 (8, 17)	25 (14, 43)	< 0.001^b*^
Plasma cTnT, median pg/ml (IQR), *N* = 412	16 (10, 26)	17 (9, 26)	18 (10, 36)	0.094^b^
Time from onset to cTnT, median months (IQR)	7 (5, 10)	13 (9, 19)	38 (19, 81)	< 0.001^b*^

^a^Pearson’s Chi-squared test

^b^Kruskal-Wallis rank sum test

^c^ALSFRS-R derived disease progression rate (DPR)

^*^Significance *p* < 0.05

### CSF NfL and plasma hs-cTnT across ALS disease aggressiveness and phases

CSF NfL levels at diagnosis were different across all three disease aggressiveness groups (*p* < 0.001), with highest concentrations observed in the high aggressiveness group (median 9 590 pg/ml) and lowest in the low aggressiveness group (median 3 375 pg/ml) (Table 1, Fig. 2A). In contrast, plasma hs-cTnT levels at diagnosis showed an opposite trend with highest values in patients with low disease aggressiveness but this difference did not reach statistical significance (Table 1, Fig. 2B).

Consistent with the group differences, we observed a significant relationship between CSF NfL and D50 in each disease aggressiveness subgroup, especially in high and low (high: *R*^2^ = 0.17, *p* < 0.001; intermediate: *R*^2^ = 0.04, *p* = 0.006; low: *R*^2^ = 0.21, *p* < 0.001; Fig. 2C), reinforcing that higher CSF NfL levels are associated with more aggressive disease. Conversely, but in line with the group comparisons, no significant association was observed between plasma cTnT levels and D50 (high: *R*^2^ = 0.0023, *p* = 0.582; intermediate: *R*^2^ = 0.012, *p* = 0.179; low: *R*^2^ = 0.0049, *p* = 0.501; Fig. 2D).

To assess the relationship with disease phase, both biomarkers were analysed in relation to rD50 at the time of sampling, which normalises disease duration to individual progression speed. Here, CSF NfL displayed only weak associations with rD50 (high: *R*^2^ = 0.025, *p* = 0.052; intermediate: *R*^2^ = 0.021, *p* = 0.047; low: *R*^2^ = 0.092, *p* = 0.002, Fig. 2E), while plasma hs**-**cTnT increased consistently with advancing rD50 (high: *R*^2^ = 0.054, *p* = 0.007; intermediate: *R*^2^ = 0.17, *p* < 0.001; low: *R*^2^ = 0.19, *p* < 0.001, Fig. 2F). Plasma hs-cTnT was not correlated to CSF NfL (*ρ* = − 0.005, *n* = 331, *p* = 0.93). In the subset of patients with paired CSF and serum samples (*n* = 109), serum and CSF NfL concentrations were strongly correlated (Pearson’s *r* = 0.77; *R*^2^ = 0.588, *p* = 2.55 × 10e-22; Supplementary Fig. 5).

### Discriminatory power of CSF NfL and plasma hs-cTnT

Logistic regression analyses were used to predict disease aggressiveness (high vs low) based on biomarker levels. In the model including CSF NfL alone, higher concentrations were associated with high disease aggressiveness (*β* = 2.79, 95% CI 2.00–3.74, *p* = 2.25e-10). In contrast, higher plasma hs-cTnT concentrations were weakly associated with low disease aggressiveness (*β* = –0.68, 95% CI − 1.28– − 0.11, *p* = 2.21e-2), similar to what was observed as a trend in the group comparisons. In the combined model including both biomarkers and their interaction term, neither biomarker remained independently significant. Details of each model is found in the Supplementary material (Supplementary Table 1).

The ROC analyses derived from the logistic regression models demonstrated strong discriminatory performance for CSF NfL (AUC 0.92) (Table 2), whereas plasma hs-cTnT achieved an AUC of 0.76. The model combining CSF NfL and plasma hs-cTnT yielded an AUC of 0.92, similar to that observed for NfL alone. Despite the absence of incremental improvement in AUC beyond NfL alone, the addition of hs-cTnT substantially altered the diagnostic classification characteristics. Sensitivity increased from 0.80 with NfL alone to 0.95 in the combined model, accompanied by a reduction in specificity (0.89–0.73). Importantly, the negative predictive value (NPV) improved markedly from 75 to 91%, while the positive predictive value remained high (84%).

**Table 2:** Discriminative ability of multivariable logistic regression models for predicting D50 low vs high

Biomarker	N	AUC	Sensitivity	Specificity	PPV	NPV
NfL	177	0.92	0.80	0.89	91%	75%
cTnT	177	0.76	0.64	0.79	82%	59%
NfL + cTnT	177	0.92	0.95	0.73	84%	91%

Area under the receiver operating characteristic curve (AUC), sensitivity, specificity, positive predictive value (PPV), and negative predictive value (NPV) are shown for each model: CSF NfL, plasma hs-cTnT, and a combined model incorporating both biomarkers. Performance metrics are derived from logistic regression analyses in the study cohort (*N* = 177). All models were adjusted for age at onset, sex, site of onset, and disease phase

Follow-up multiple linear regression analyses assessed the association between biomarker levels and continuous D50. The model comprising only CSF NfL accounted for 34.8% of the variance in D50, with RMSE 0.57 and AIC 774.65 (Supplementary Fig.  1 A). The model including only plasma hs-cTnT accounted for 0.2% of the variance, with RMSE 0.79 and AIC 911.57 (Supplementary Fig. 1B). The combined model accounted for 25.8% of the variance, with RMSE 0.60 and AIC 555.98 (Supplementary Fig.  1 C). Although the combined model explained a smaller proportion of variance than CSF NfL alone, it yielded the lowest AIC and a comparable RMSE, indicating an overall better model fit when both biomarkers were included.

Together, these analyses support that while CSF NfL remains the strongest single predictor of disease aggressiveness, inclusion of plasma hs-cTnT further refines the prediction.

### Associations to site of symptom onset

**Fig. 3:**
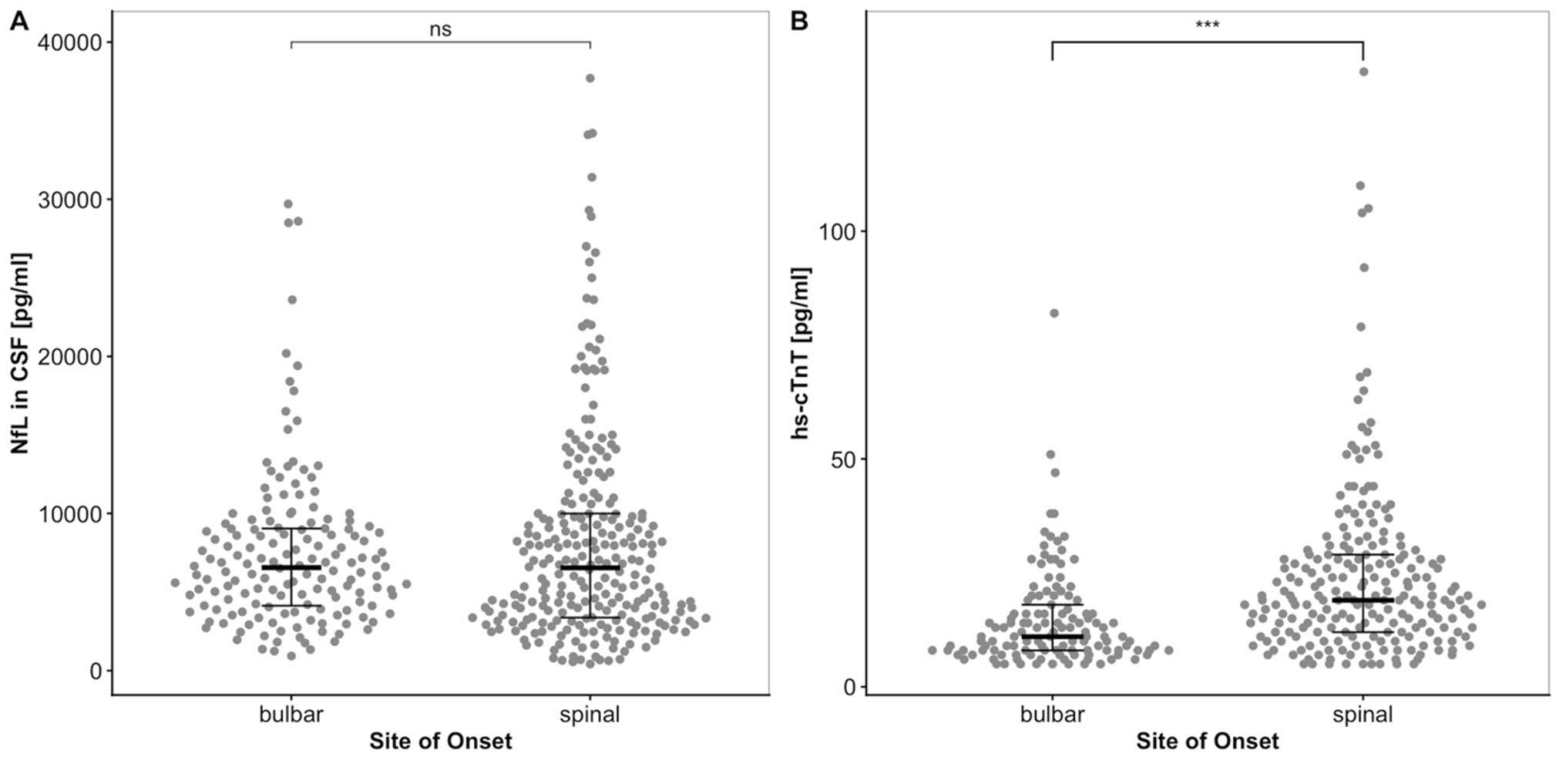
NfL and cTnT by site of onset (*n* = 475). **A** NfL in CSF by site of symptom onset. **B** Plasma troponin (hs-cTnT) by site of symptom onset. Wilcoxon-Rank-Sum-Test: ns = non-significant; * = *p* < 0.05; ** = *p* < 0.01; *** = *p* < 0.001; median values are indicated as horizontal lines and IQR as error bars

Group comparisons based on site of symptom onset (bulbar vs spinal) revealed lower plasma hs-cTnT levels in patients with bulbar onset compared to spinal (*p* < 0.001) (Fig. 3). This pattern was not observed for CSF NfL, where levels did not differ by site of onset. To further explore potential site-specific relationships, we performed stratified linear regression analyses of biomarker levels against disease aggressiveness and disease phase, separately for bulbar and spinal onset (Supplementary Fig. 2). For CSF NfL, a negative association with aggressiveness was observed across both onset sites (Supplementary Fig.  2 A). For plasma hs-cTnT, the association with aggressiveness was somewhat different than for CSF NfL: in bulbar onset patients, plasma hs-cTnT showed a weak negative association with aggressiveness, whereas in spinal onset patients, the relationship appeared non-linear, with slightly higher concentrations in the intermediate aggressiveness group and lower values in both high and low aggressiveness groups (Supplementary Fig. 2B). When analysing disease phase, both CSF NfL and hs-cTnT displayed similar patterns between spinal and bulbar onset patients (Supplementary Fig.  2 A and B).

Next, we performed logistic regression and ROC curve analyses to evaluate whether the biomarkers could discriminate between sites of symptom onset (bulbar vs. spinal). Interestingly, plasma hs-cTnT was independently associated with site of symptom onset, whereas CSF NfL was not (Supplementary Table 2). Even if the prediction of site of symptom onset in general was less accurate than for disease aggressiveness, plasma hs-cTnT demonstrated comparatively higher AUC compared with CSF NfL (0.75 for plasma hs-cTnT alone, 0.71 for CSF NfL alone, and 0.75 for the combined model).

## Discussion

In this population-based cohort of patients with ALS, we confirm previous evidence that plasma hs-cTnT is elevated at the time of diagnosis, alongside CSF NfL. Although both biomarkers were elevated in the ALS population, they did not correlate, suggesting that they reflect distinct pathophysiological processes. Consistent with earlier work [15], we also confirmed significant differences in CSF NfL levels between D50-derived disease aggressiveness subgroups, with higher CSF NfL concentrations in patients with more aggressive disease. In contrast, plasma hs-cTnT levels did not differ significantly between disease aggressiveness subgroups, although a trend toward higher concentrations in the less aggressive group was observed. Elevated plasma hs-cTnT concentrations were frequently observed in our ALS cohort, consistent with prior reports of skeletal muscle-derived release [3, 5, 7, 18]. These findings suggest that the biological sources of plasma hs-cTnT release differ from those of CSF NfL, and that muscle-related processes may underlie its elevation in ALS.

Analyses across the rD50 generated disease trajectory, further supported distinct temporal dynamics for the two biomarkers. CSF NfL behaved uniformly across all disease aggressiveness subgroups, showing a consistent positive association with disease phase (rD50) independent of aggressiveness. This pattern, characterised by overlapping trajectories even in patients with lower CSF NfL values, underscores that NfL retains predictive utility across the full disease spectrum and remains suitable for clinical trial stratification independent of disease phase or rate. Conversely, plasma hs-cTnT showed a different slope across rD50, with relatively greater increases in patients with slower progression, possibly reflecting the influence of preserved muscle mass or more gradual muscle involvement [19, 20]. These divergent rD50 patterns support the hypothesis that CSF NfL primarily reflects the rate of neuronal loss (disease aggressiveness), whereas plasma hs-cTnT may capture peripheral processes related to muscle denervation, spread, or cumulative disease burden. A related but somewhat alternative interpretation is that patients with slower disease trajectories spend longer time in a state of active denervation-reinnervation and muscle remodelling, allowing greater cumulative troponin release over time. In that sense, hs-cTnT may be less a marker of instantaneous damage intensity than of the duration and topographic extent of muscle involvement [19].

The logistic regression and ROC curve analyses demonstrated moderate to strong discriminatory performance predicting high vs low disease aggressiveness. Although the addition of plasma hs-cTnT to CSF NfL did not improve overall discrimination as assessed by AUC, it altered key classification characteristics. In particular, the combined model improved sensitivity and negative predictive value, thereby strengthening the ability to identify patients with less aggressive disease. Interestingly, plasma hs-cTnT demonstrated higher discriminatory performance than CSF NfL in differentiating between sites of symptom onset. Patients with spinal onset exhibited higher plasma hs-cTnT levels than those with bulbar onset, indicating that troponin release may depend on disease topography or the extent of muscles involved, similar to what was observed in Chamoun et al., 2025 [8]. Since spinal onset ALS typically affects larger proximal muscle groups compared to bulbar onset, elevated plasma hs-cTnT in this subgroup may reflect greater muscle turnover in regions with higher overall muscle mass. This classification accuracy of plasma hs-cTnT suggests utility as a complementary biomarker of clinical phenotype, capturing peripheral aspects of disease biology that are not reflected by CSF NfL. Even though our study design cannot determine tissue origin, a couple of candidate mechanisms deserve consideration. Circulating hs-cTnT may arise either from active skeletal muscle remodelling, including fibre-type switching and dysregulated muscle regeneration, or from passive leakage due to myofibre damage and breakdown. This distinction matters clinically: a biomarker generated by active remodelling would be expected to accumulate with disease phase and topographic spread, as observed in our study, whereas a purely passive leakage marker might be expected to track more closely with acute tissue destruction or aggressive decline. These mechanistic considerations are in line with prior work in ALS muscle showing activation of muscle regeneration pathways in ALS despite incomplete repair, a setting in which sarcomeric proteins may be produced, turned over, and released without stable incorporation into mature fibres [21, 22].

Recent findings by Bernsen et al. (2025) [23] have highlighted the clinical relevance of troponin, identifying it as a candidate marker for therapeutic response in patients treated with tofersen. Our interpretation of plasma hs-cTnT as a marker of cumulative peripheral involvement in ALS implies that a therapy which slows or stabilises motor neuron loss would be hypothesised to attenuate this process and thereby reduce circulating troponin levels [18]. While NfL is currently the most established biomarker of neuroaxonal injury in ALS and has emerged as an important pharmacodynamic biomarker in therapeutic trials, its role as a surrogate endpoint remains an area of active discussion, as longitudinal NfL concentrations are generally stable in untreated disease despite ongoing functional decline. hs-cTnT may provide complementary information on downstream skeletal muscle denervation, remodelling, and preservation. This distinction may be particularly relevant for therapies targeting muscle or the neuromuscular unit, where hs-cTnT may capture treatment effects not reflected by NfL alone. Furthermore, the strong correlation between paired serum and CSF NfL supports the feasibility of future trial designs combining serum NfL and plasma hs-cTnT from a single blood sample to capture complementary aspects of ALS pathology. Taken together, this strengthens the view of troponin as a relevant biomarker in ALS. Future studies should clarify the molecular origin of circulating troponin in ALS. It will also be important to examine whether elevated troponin levels in spinal onset ALS are linked to respiratory functional decline or cardiac strain secondary to chronic respiratory compromise. However, the repeatedly observed pattern of elevated cTnT with normal cTnI makes major myocardial injury an unlikely sole explanation in most patients, although mixed contributions in advanced disease cannot be excluded [5, 7].

The strengths of this study include the population-based design, the large and well-characterised cohort, and the parallel evaluation of troponin and NfL. However, the absence of systematic cardiac assessments limits the ability to fully exclude cardiac contributions to plasma hs-cTnT elevations. Further studies integrating cardiac evaluations and muscle-specific troponin isoform profiling are warranted to elucidate the tissue origins and temporal dynamics of troponin release in ALS. In addition, the study’s single-center, observational design may limit generalisability, and restricts interpretation of troponin dynamics beyond associative relationships. The predictive performance of the logistic regression models was evaluated within the present cohort and should therefore be considered exploratory until validated in independent ALS populations. Finally, CSF NfL and plasma hs-cTnT were not always obtained at the same sampling occasion. However, both biomarkers were generally sampled during the diagnostic work-up, and restricting analyses to samples obtained within ± 30 days did not materially alter the results. Nevertheless, some additional variability, particularly in rapidly progressive patients, cannot be excluded.

In conclusion, our findings show that plasma hs-cTnT and CSF NfL provide complementary but biologically distinct information in ALS. Whereas CSF NfL reliably reflects neuronal injury and disease aggressiveness, plasma hs-cTnT appears to index processes related to disease accumulation. These complementary roles underscore the potential of integrating both biomarkers to improve disease stratification, mechanistic understanding, and monitoring of therapeutic response in ALS.

## Supplementary Information

Below is the link to the electronic supplementary material.

Supplementary file1 (DOCX 14625 KB)

## Data Availability

The data supporting the conclusion of this article will be made available by the authors upon reasonable request.

## Abbreviations

AIC: Akaike information criterion
ALS: Amyotrophic lateral sclerosis
ALSFRS-R: Revised ALS functional rating scale
AUC: Area under the ROC curve
C9orf72: Chromosome 9 open reading frame 72
cFL: Calculated functional loss rate
cFS: Calculated functional state
CSF: Cerebrospinal fluid
D50: Global disease aggressiveness
DPR: Derived progression rate
dx: Time constant of functional decline
ELISA: Enzyme-linked immunosorbent assay
hs-cTnT: High sensitive cardiac troponin T
IQR: Interquartile range
NfL: Neurofilament light chain
NPV: Negative predictive value
PPV: Positive predictive value
rD50: Relative disease phase
ROC: Receiver operating characteristic
RMSE: Root mean square error
SOD1: Superoxide dismutase 1

## References

[1] Bridel C, van Wieringen WN, Zetterberg H, Tijms BM, Teunissen CE, and the NFL Group (2019) Diagnostic value of cerebrospinal fluid neurofilament light protein in neurology: a systematic review and meta-analysis. JAMA Neurol 76(1035):1048. 10.1001/jamaneurol.2019.1534 10.1001/jamaneurol.2019.1534 PMC6580449 31206160

[2] Olofsson J, Bergström S, Mravinacová S, Kläppe U, Öijerstedt L, Zetterberg H, Blennow K, Ingre C, Nilsson P, Månberg A (2025) Cerebrospinal fluid levels of NfM in relation to NfL and pNfH as prognostic markers in amyotrophic lateral sclerosis. Amyotroph Later Scler Frontotemporal Degener 26:113–123. 10.1080/21678421.2024.2428930 10.1080/21678421.2024.2428930 39575564

[3] Kläppe U, Chamoun S, Shen Q, Finn A, Evertsson B, Zetterberg H, Blennow K, Press R, Samuelsson K, Månberg A, Fang F, Ingre C (2022) Cardiac troponin T is elevated and increases longitudinally in ALS patients. Amyotroph Later Scler Frontotemporal Degener 23:58–65. 10.1080/21678421.2021.1939384 10.1080/21678421.2021.1939384 34151677

[4] Meyer T, Schumann P, Weydt P, Petri S, Koc Y, Spittel S, Bernsen S, Günther R, Weishaupt JH, Dreger M, Kolzarek F, Kettemann D, Norden J, Boentert M, Vidovic M, Meisel C, Münch C, Maier A, Körtvélyessy P (2023) Neurofilament light-chain response during therapy with antisense oligonucleotide tofersen in SOD1-related ALS: treatment experience in clinical practice. Muscle Nerve 67:515–521. 10.1002/mus.27818 36928619 10.1002/mus.27818

[5] Rittoo D, Jones A, Lecky B, Neithercut D (2014) Elevation of cardiac troponin T, but not cardiac troponin I, in patients with neuromuscular diseases. J Am Coll Cardiol 63:2411–2420. 10.1016/j.jacc.2014.03.027 24747102 10.1016/j.jacc.2014.03.027

[6] du de Fay Lavallaz J, Prepoudis A, Wendebourg MJ, Kesenheimer E, Kyburz D, Daikeler T, Haaf P, Wanschitz J, Löscher WN, Schreiner B, Katan M, Jung HH, Maurer B, Hammerer-Lercher A, Mayr A, Gualandro DM, Acket A, Puelacher C, Boeddinghaus J, Nestelberger T, Lopez-Ayala P, Glarner N, Shrestha S, Manka R, Gawinecka J, Piscuoglio S, Gallon J, Wiedemann S, Sinnreich M, Mueller C, for the BASEL XII Investigators (2022) Skeletal muscle disorders: a noncardiac source of cardiac troponin T. Circulation 145:1764–1779. 10.1161/CIRCULATIONAHA.121.058489 35389756 10.1161/CIRCULATIONAHA.121.058489 PMC10069758

[7] Castro-Gomez S, Radermacher B, Tacik P, Mirandola SR, Heneka MT, Weydt P (2021) Teaching an old dog new tricks: serum troponin T as a biomarker in amyotrophic lateral sclerosis. Brain Commun 3:fcab274. 10.1093/braincomms/fcab274 34993474 10.1093/braincomms/fcab274 PMC8728713

[8] Chamoun S, Imrell S, Upate Z, Kläppe U, Öijerstedt L, Yazdani S, Andersson Franko M, Foucher J, Azizi L, Lovik A, Samuelsson K, Press R, Fang F, Svennberg E, Juto A, Ingre C (2025) Plasma troponin T reflects lower motor neuron involvement on electromyography in amyotrophic lateral sclerosis. Brain Commun 7:fcaf177. 10.1093/braincomms/fcaf177 40385376 10.1093/braincomms/fcaf177 PMC12082033

[9] Dergai O, Wuu J, Koziczak-Holbro M, Malaspina A, Granit V, Hernandez JP, Cooley A, Sachdev R, Yu L, Bidinosti M, Flotte L, Nash M, Jennings LL, Berry JD, Bruijn LI, Brachat S, Benatar M (2026) Skeletal muscle biomarkers of amyotrophic lateral sclerosis: a large-scale, multi-cohort proteomic study. Ann Neurol 99:393–407. 10.1002/ana.78046 41020397 10.1002/ana.78046 PMC12894501

[10] Poesen K, De Schaepdryver M, Stubendorff B, Gille B, Muckova P, Wendler S, Prell T, Ringer TM, Rhode H, Stevens O, Claeys KG, Couwelier G, D’Hondt A, Lamaire N, Tilkin P, Van Reijen D, Gourmaud S, Fedtke N, Heiling B, Rumpel M, Rödiger A, Gunkel A, Witte OW, Paquet C, Vandenberghe R, Grosskreutz J, Van Damme P (2017) Neurofilament markers for ALS correlate with extent of upper and lower motor neuron disease. Neurology 88:2302–2309. 10.1212/WNL.0000000000004029 28500227 10.1212/WNL.0000000000004029

[11] Prell T, Gaur N, Steinbach R, Witte OW, Grosskreutz J (2020) Modelling disease course in amyotrophic lateral sclerosis: pseudo-longitudinal insights from cross-sectional health-related quality of life data. Health Qual Life Outcomes 18:117. 10.1186/s12955-020-01372-6 32357946 10.1186/s12955-020-01372-6 PMC7195704

[12] Meyer J, Gaur N, Von Der Gablentz J, Friedrich B, Roediger A, Grosskreutz J, Steinbach R (2025) Phosphorylated neurofilament heavy chain (pNfH) concentration in cerebrospinal fluid predicts overall disease aggressiveness (D50) in amyotrophic lateral sclerosis. Front Neurosci 19:1536818. 10.3389/fnins.2025.1536818 40143847 10.3389/fnins.2025.1536818 PMC11936903

[13] Gaur N, Steinbach R, Plaas M, Witte OW, Brill MS, Grosskreutz J (2023) Chitinase dysregulation predicts disease aggressiveness in ALS: insights from the D50 progression model. J Neurol Neurosurg Psychiatry 94:585–588. 10.1136/jnnp-2022-330318 37076292 10.1136/jnnp-2022-330318

[14] Magen I, Yacovzada NS, Yanowski E, Coenen-Stass A, Grosskreutz J, Lu C-H, Greensmith L, Malaspina A, Fratta P, Hornstein E (2021) Circulating miR-181 is a prognostic biomarker for amyotrophic lateral sclerosis. Nat Neurosci 24:1534–1541. 10.1038/s41593-021-00936-z 34711961 10.1038/s41593-021-00936-z

[15] Dreger M, Steinbach R, Gaur N, Metzner K, Stubendorff B, Witte OW, Grosskreutz J (2021) Cerebrospinal fluid neurofilament light chain (NfL) predicts disease aggressiveness in amyotrophic lateral sclerosis: an application of the D50 disease progression model. Front Neurosci 15:651651. 10.3389/fnins.2021.651651 33889072 10.3389/fnins.2021.651651 PMC8056017

[16] Steinbach R, Gaur N, Roediger A, Mayer TE, Witte OW, Prell T, Grosskreutz J (2021) Disease aggressiveness signatures of amyotrophic lateral sclerosis in white matter tracts revealed by the D50 disease progression model. Hum Brain Mapp 42:737–752. 10.1002/hbm.25258 33103324 10.1002/hbm.25258 PMC7814763

[17] Steinbach R, Batyrbekova M, Gaur N, Voss A, Stubendorff B, Mayer TE, Gaser C, Witte OW, Prell T, Grosskreutz J (2020) Applying the D50 disease progression model to gray and white matter pathology in amyotrophic lateral sclerosis. Neuroimage Clin 25:102094. 10.1016/j.nicl.2019.102094 31896467 10.1016/j.nicl.2019.102094 PMC6940701

[18] Lindenborn P, Fabian R, Grehl T, Nazlican H, Meyer T, Bernsen S, Weydt P (2026) Combination of serum neurofilament light chain and serum cardiac troponin T as biomarkers improves diagnostic accuracy in amyotrophic lateral sclerosis. Ann Neurol 99:408–417. 10.1002/ana.78066 41133969 10.1002/ana.78066 PMC12894507

[19] Duranti E (2025) The role of skeletal muscle in amyotrophic lateral sclerosis: state of the art 2025. Muscles (Basel) 4:22. 10.3390/muscles4030022

[20] Vielhaber S, Winkler K, Kirches E, Kunz D, Büchner M, Feistner H, Elger CE, Ludolph AC, Riepe MW, Kunz WS (1999) Visualization of defective mitochondrial function in skeletal muscle fibers of patients with sporadic amyotrophic lateral sclerosis. J Neurol Sci 169:133–139. 10.1016/S0022-510X(99)00236-1 10540022 10.1016/s0022-510x(99)00236-1

[21] Jensen L, Jørgensen LH, Bech RD, Frandsen U, Schrøder HD (2016) Skeletal muscle remodelling as a function of disease progression in amyotrophic lateral sclerosis. Biomed Res Int 2016:5930621. 10.1155/2016/5930621 27195289 10.1155/2016/5930621 PMC4852332

[22] Manzano R, Toivonen JM, Oliván S, Calvo AC, Moreno-Igoa M, Muñoz MJ, Zaragoza P, García-Redondo A, Osta R (2011) Altered expression of myogenic regulatory factors in the mouse model of amyotrophic lateral sclerosis. Neurodegener Dis 8:386–396. 10.1159/000324159 21346327 10.1159/000324159

[23] Bernsen S, Fabian R, Koc Y, Schumann P, Körtvélyessy P, Castro-Gomez S, Meyer T, Weydt P (2025) Serum cardiac troponin T levels as a therapy response marker in tofersen-treated ALS. Muscle Nerve. 10.1002/mus.28453 40491248 10.1002/mus.28453 PMC12338008

