# Supplementary material for: Plasma cardiac troponin T complements neurofilament light chain by reflecting disease phase and phenotypic variation in amyotrophic lateral sclerosis
Source: J Neurol. 2026 Jul 27;273(8):494. doi: 10.1007/s00415-026-14002-w (PMC13407708; doi:10.1007/s00415-026-14002-w)
Supplement: Supplementary file 1 — Supplementary file1 (DOCX 14625 KB) [file 415_2026_14002_MOESM1_ESM.docx]

# SUPPLEMENTARY MATERIALS

Plasma cardiac troponin T complements neurofilament light chain by reflecting disease phase and phenotypic variation in amyotrophic lateral sclerosis

Sellin and Öijerstedt et al. 2026

**Supplementary Table 1.** Logistic regression models (low vs high aggressiveness, low is reference):

| **Variable** | **Estimate (log-odds)** | **CI** | **Std. Error** | **P-value** |
| --- | --- | --- | --- | --- |
| NfL | | | | |
| Intercept | -27.32 | -36.79–-19.59 | 4.35 | 3.54E-10 |
| NfL | 2.79 | 2.00–3.74 | 0.44 | 2.25E-10 |
| Bulbar onset | 0.94 | -0.02–1.95 | 0.5 | 0.060657 |
| Male sex | 0.88 | -0.06–1.87 | 0.49 | 0.071038 |
| Age at onset | 0.04 | 0.00–0.09 | 0.02 | 0.054845 |
| Phase II | -0.05 | -1.08–0.94 | 0.51 | 0.91984 |
| Phase III/IV | -0.24 | -2.08–1.90 | 0.98 | 0.80519 |
| cTnT | | | | |
| Intercept | -1.5 | -3.76–0.68 | 1.13 | 0.181763 |
| cTnT | -0.68 | -1.28–-0.11 | 0.3 | 0.02205 |
| Bulbar onset | 0.52 | -0.24–1.31 | 0.39 | 0.187666 |
| Male sex | 0.95 | 0.22–1.72 | 0.38 | 0.012628 |
| Age at onset | 0.04 | 0.01–0.07 | 0.02 | 0.023222 |
| Phase II | 1.39 | 0.67–2.15 | 0.38 | 0.000218 |
| Phase III/IV | 1.68 | 0.10–3.70 | 0.88 | 0.056095 |
| NfL + cTnT | | | | |
| Intercept | -21.24 | -53.48–7.73 | 15.65 | 0.17463 |
| cTnT | -2.22 | -13.43–8.51 | 5.62 | 0.692713 |
| NfL | 2.09 | -1.25–5.77 | 1.79 | 0.242739 |
| Bulbar onset | 0.91 | -0.08–1.96 | 0.52 | 0.077887 |
| Male sex | 0.89 | -0.09–1.93 | 0.51 | 0.080197 |
| Age at onset | 0.04 | 0.00–0.09 | 0.02 | 0.054838 |
| Phase II | -0.03 | -1.10–0.99 | 0.53 | 0.949897 |
| Phase III/IV | -0.24 | -2.17–1.99 | 1.03 | 0.81849 |
| cTnT:NfL | 0.25 | -0.99–1.54 | 0.65 | 0.697931 |

**Supplementary Table 2.** Logistic regression models (bulbar vs spinal, bulbar is reference):

| **Variable** | **Estimate (log-odds)** | **CI** | **Std. Error** | **P-value** |
| --- | --- | --- | --- | --- |
| NfL | | | | |
| Intercept | -0.27 | -5.34–4.90 | 2.6 | 0.918118 |
| NfL | 0.24 | -0.28–0.77 | 0.27 | 0.362655 |
| Low aggressiveness | 1.02 | 0.08–2.00 | 0.49 | 0.036665 |
| Male sex | 0.95 | 0.29–1.64 | 0.34 | 0.005354 |
| Age at onset | -0.03 | -0.07–-0.00 | 0.02 | 0.03588 |
| Phase II | 0.25 | -0.55–1.07 | 0.41 | 0.542116 |
| Phase III/IV | -0.49 | -1.96–0.98 | 0.74 | 0.50163 |
| cTnT | | | | |
| Intercept | 0.71 | -1.60–3.06 | 1.18 | 0.547676 |
| cTnT | 0.92 | 0.33–1.57 | 0.31 | 0.003345 |
| Low aggressiveness | 0.56 | -0.20–1.35 | 0.39 | 0.153215 |
| Male sex | 0.63 | -0.08–1.35 | 0.36 | 0.08194 |
| Age at onset | -0.05 | -0.09–-0.02 | 0.02 | 0.00566 |
| Phase II | 0.21 | -0.57–1.01 | 0.4 | 0.592113 |
| Phase III/IV | -0.88 | -2.40–0.63 | 0.76 | 0.244607 |
| NfL + cTnT | | | | |
| Intercept | -1.43 | -14.75–11.42 | 6.58 | 0.827629 |
| cTnT | 0.4 | -4.37–5.46 | 2.48 | 0.870601 |
| NfL | 0.23 | -1.25–1.75 | 0.76 | 0.765824 |
| Low aggressiveness | 1 | 0.04–2.01 | 0.5 | 0.045919 |
| Male sex | 0.6 | -0.11–1.32 | 0.36 | 0.100313 |
| Age at onset | -0.05 | -0.09–-0.02 | 0.02 | 0.004603 |
| Phase II | -0.01 | -0.85–0.84 | 0.43 | 0.987934 |
| Phase III/IV | -1.11 | -2.71–0.45 | 0.79 | 0.160555 |
| cTnT:NfL | 0.07 | -0.51–0.63 | 0.29 | 0.808733 |

Non-parametric tests:

|  | Plasma hc-TnT | CSF NfL |
| --- | --- | --- |
| D50 high-intermediate | 1.00 | 1.2e-11 |
| D50 high-low | 0.19 | <2e-16 |
| D50 intermediate-low | 0.13 | 5.2e-08 |
| Site of onset bulbar-spinal | 1.17e-8 | 1.00 |
| Wilcoxon Rank sum test | | |


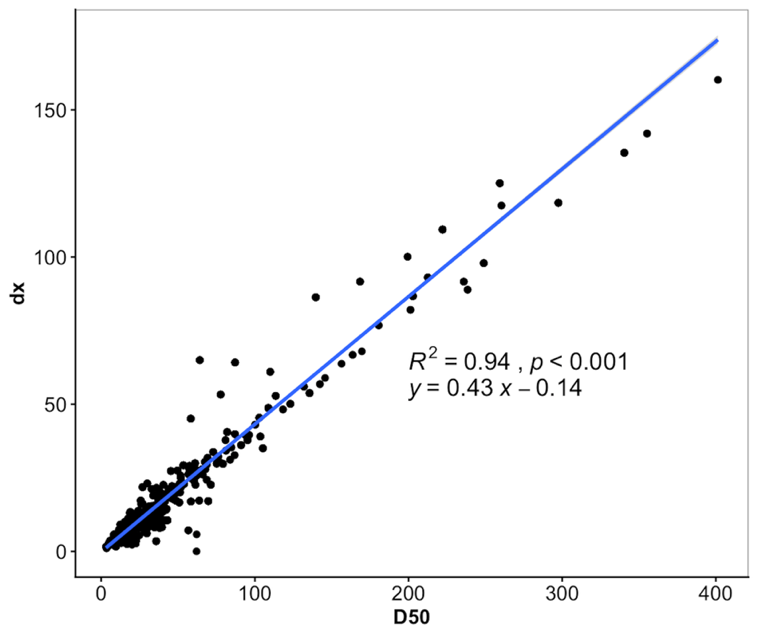


**Supplementary Figure 1. Correlation of dx and D50.** D50 in months correlates highly with dx, which is the time constant of functional decline.


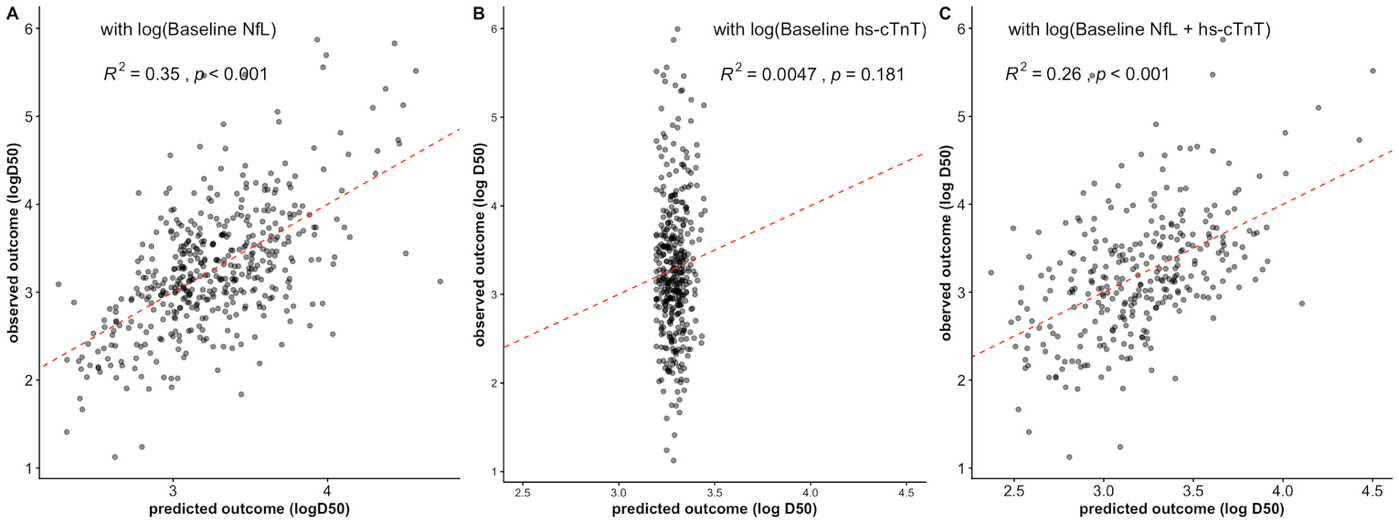
**Supplementary Figure 2. Linear regression models predicting disease aggressiveness from baseline biomarker levels**. Scatter plots show the relationship between predicted and observed log D50 for models including (**A**) CSF NfL, (**B**) plasma hs-cTnT, and (**C**) both biomarkers combined.


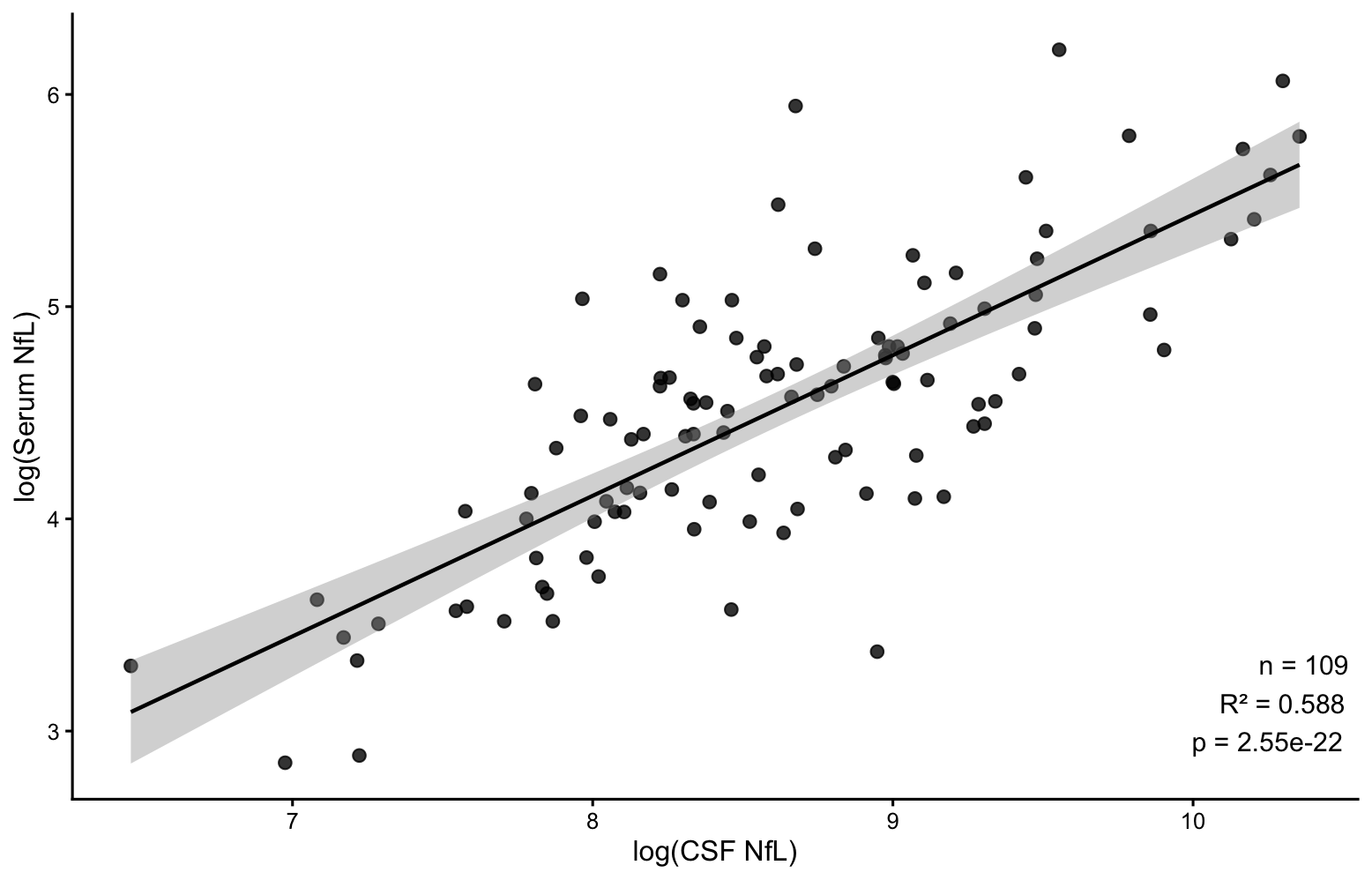


**Supplementary Figure 3.** **Association between CSF NfL and serum NfL**. Each point represents one individual (N=109). The black line represents the fitted linear regression line, with the shaded area indicating the 95% confidence interval. The displayed R² and p-value are from a linear regression model with log(serum NfL) as the dependent variable and log(CSF NfL) as the independent variable. NfL, neurofilament light chain; CSF, cerebrospinal fluid.


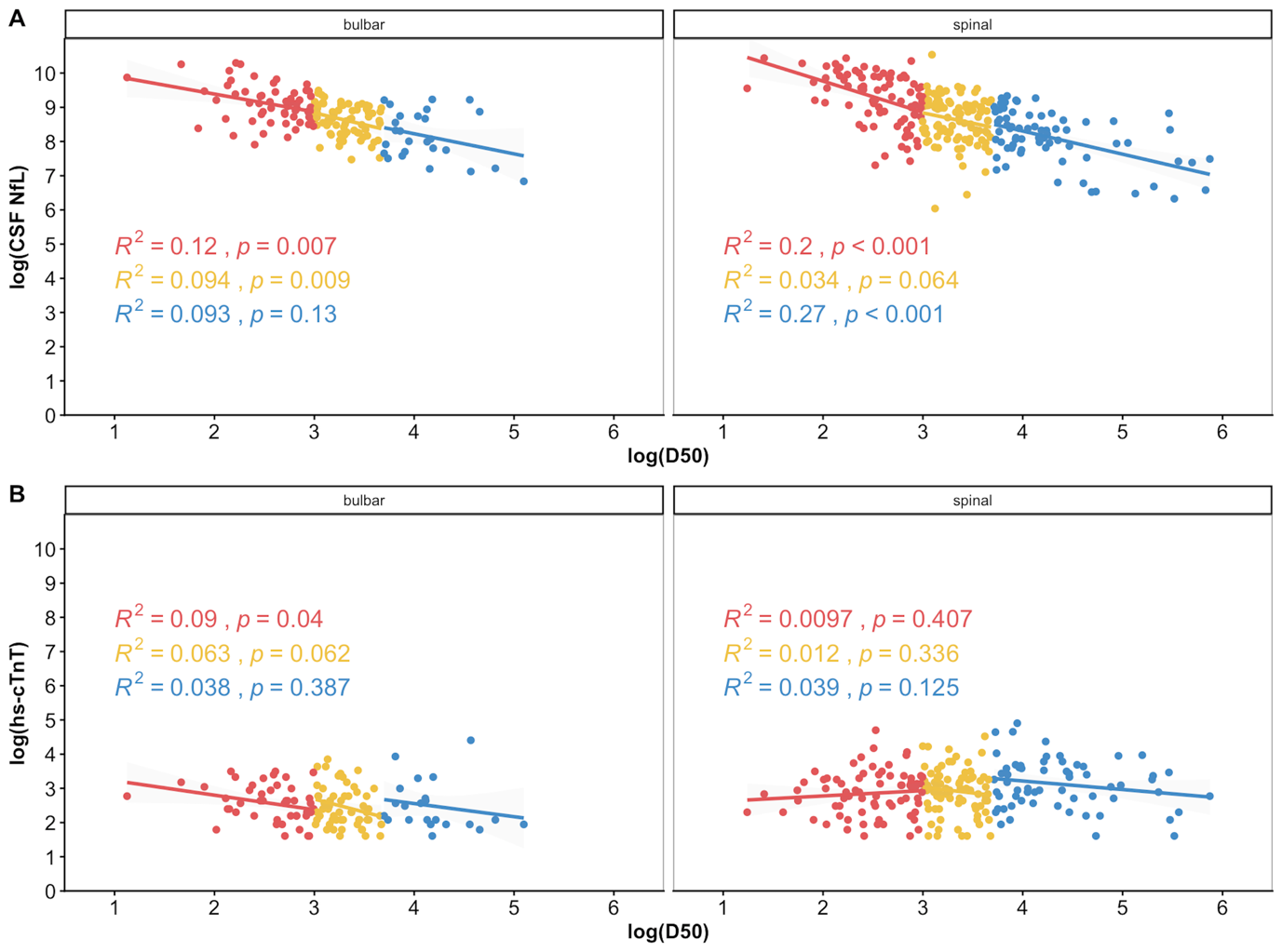
**Supplementary Figure 4. Correlation of D50, biomarkers, disease aggressiveness and onset region.** Linear regression with log(D50) and **(A)** log(NfL) and **(B)** log(hs-cTnT), both divided into symptom onset regions bulbar and spinal.


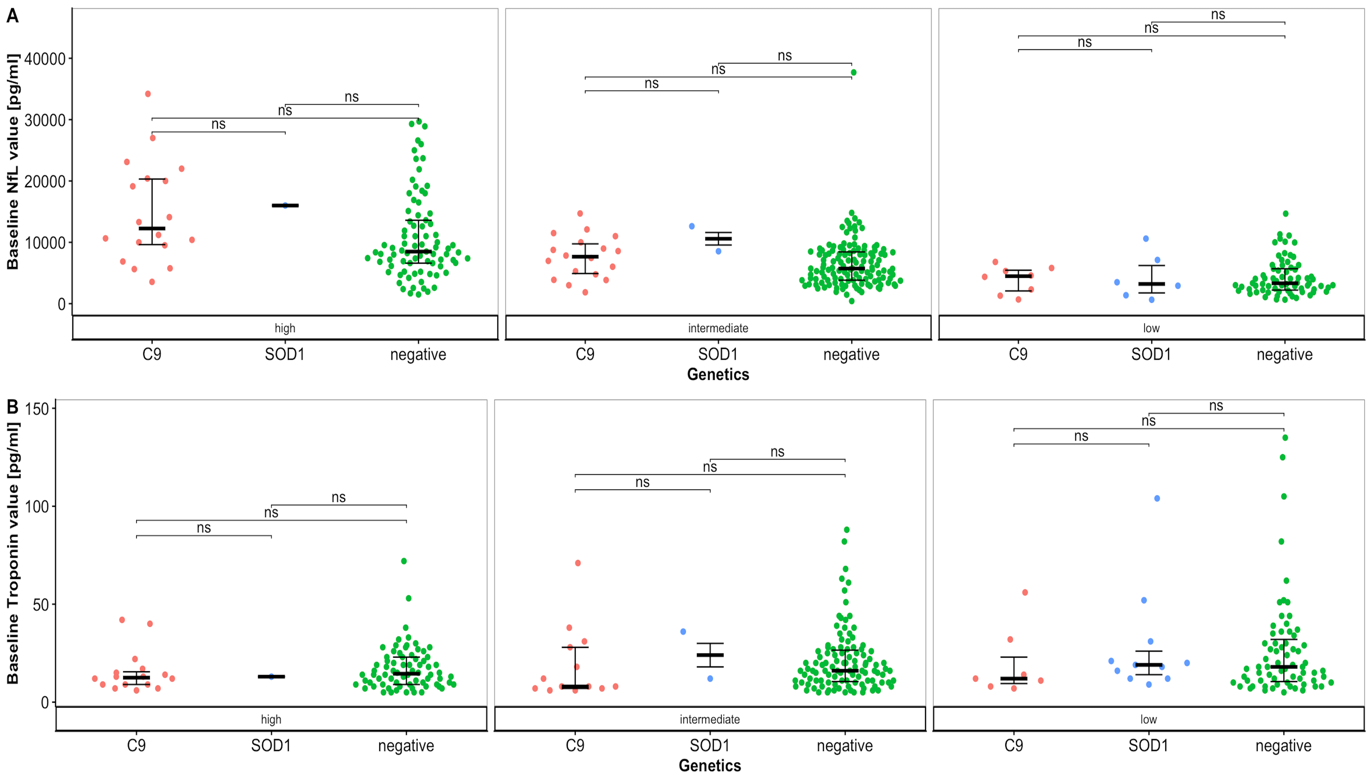


**Supplementary Figure 5.** Relationship between (**A**) CSF NfL and (**B**) plasma hs-cTnT and known genetic status, divided into D50-derived disease aggressiveness; median values are indicated as horizontal lines and IQR as error bars, Wilcoxon-Rank-Sum-Test: ns = non-significant; colors: D50-derived disease aggressiveness, red = high (D50 ≤ 20 months), yellow= intermediate (20 < D50 < 40 months), blue = low aggressive disease (D50 ≥ 40 months).
